# *Mycoplasma agassizii*, an opportunistic pathogen of tortoises, shows very little genetic variation across the Mojave and Sonoran Deserts

**DOI:** 10.1371/journal.pone.0245895

**Published:** 2021-02-03

**Authors:** Agusto Luzuriaga-Neira, Franziska C. Sandmeier, Chava L. Weitzman, C. Richard Tracy, Shalyn N. Bauschlicher, Richard L. Tillett, David Alvarez-Ponce

**Affiliations:** 1 Department of Biology, University of Nevada Reno, Reno, Nevada, United States of America; 2 Biology Department, Colorado State University, Pueblo, Colorado, United States of America; 3 Department of Biological Sciences, Virginia Polytechnic Institute, Blacksburg, Virginia, United States of America; 4 Nevada Center for Bioinformatics, University of Nevada, Reno, Nevada, United States of America; Academia Sinica, TAIWAN

## Abstract

*Mycoplasma agassizii* is a common cause of upper respiratory tract disease in Mojave desert tortoises (*Gopherus agassizii*). So far, only two strains of this bacterium have been sequenced, and very little is known about its patterns of genetic diversity. Understanding genetic variability of this pathogen is essential to implement conservation programs for their threatened, long-lived hosts. We used next generation sequencing to explore the genomic diversity of 86 cultured samples of *M*. *agassizii* collected from mostly healthy Mojave and Sonoran desert tortoises in 2011 and 2012. All samples with enough sequencing coverage exhibited a higher similarity to *M*. *agassizii* strain PS6^T^ (collected in Las Vegas Valley, Nevada) than to strain 723 (collected in Sanibel Island, Florida). All eight genomes with a sequencing coverage over 2x were subjected to multiple analyses to detect single-nucleotide polymorphisms (SNPs). Strikingly, even though we detected 1373 SNPs between strains PS6^T^ and 723, we did not detect any SNP between PS6^T^ and our eight samples. Our whole genome analyses reveal that *M*. *agassizii* strain PS6^T^ may be present across a wide geographic extent in healthy Mojave and Sonoran desert tortoises.

## Introduction

Epidemiological theory offers a basic framework to ask questions about the evolution and genetic diversity of pathogens across the landscape. In particular, highly virulent pathogens in vertebrate hosts may show little genetic diversity due to a new, clonal strain sweeping through a population in an epidemic [1]. When pathogens lead to chronic, non-fatal disease, they often are not subject to intense pressure from the immune system and therefore may acquire more genetic diversity by drift [1]. Both of these models assume strong adaptive immune responses when the immune system is activated. However, ectothermic animals often do not show strong adaptive immunity, and genetic diversity of virulent, as well as chronic, pathogens may be the rule instead of the exception [2]. Furthermore, different microbes may also have very different evolutionary patterns. For example, mycoplasmas (Mollicutes) include both pathogenic and nonpathogenic species, and evolution across the group is marked by reduction of the genome and horizontal gene transfer [3].

Epidemiological research has shown that evolution and genetic diversity may vary drastically among host–mycoplasma systems. For example, when *Mycoplasma gallisepticum* was first introduced to wild passerine populations, selection appeared to favor increasing virulence [4]. In contrast, the human pathogen *Mycoplasma pneumoniae* shows fewer genetic differences among strains, despite a global distribution [5]. Mycoplasmal diseases provide excellent systems for studying genetic diversity of pathogens, as they often exhibit naturally high mutation rates, likely due to a reduction of repair mechanisms for DNA [6,7].

Here, we investigate the genetic diversity of a mycoplasma, *Mycoplasma agassizii*, that causes upper respiratory tract disease (URTD) in Mojave desert tortoises (*Gopherus agassizii*) in the USA. While *M*. *agassizii* can occur at very high frequencies in some populations, it only causes disease when it reaches high loads within animals [8,9]. Tortoises can mount an adaptive immune response to *M*. *agassizii*, but it is not effective at clearing the pathogen and can result in recrudescence of the disease [10]. Such patterns of chronic disease are not unique to tortoises and occur in many other vertebrate animals due to diseases caused by mycoplasmas [11]. However, how tortoises reduce loads of silent infections is currently unknown, but likely involves innate immunity and is possibly mediated by innate B1 lymphocytes and natural antibodies [12]. We have an incomplete understanding of the factors leading to increased infection intensity and disease, and whether disease is driven primarily by host immune function or by differences in the strains of *M*. *agassizii*. Conserved regions of the *M*. *agassizii* genome (16S rRNA, the 16-23S intergenic spacer region, and RNA polymerase ß subunit) showed some single nucleotide polymorphisms (SNPs), but very low diversity across the range of the Mojave desert [13].

One problem in understanding this disease in wild animals is that *M*. *agassizii* is hard to culture, and researchers currently use liquid cultures in SP4 broth, which can require multiple weeks after isolation from tortoises to grow to mid-log phase [14]. In addition, isolation rates of the microbe from tortoises are inconsistent, and liquid culturing of mucosal samples that contain many different types of bacteria often results in some contamination (Sandmeier et al., unpublished data). However, during the course of a wide-ranging survey of disease in desert tortoises in 2010–2012 [9,13], we used a modified technique to attempt to culture *M*. *agassizii* from 106 animals, representing 12 populations of Mojave desert tortoises and 2 populations of Sonoran desert tortoises (*Gopherus morafkai*).

To quantify genetic differences among strains, we used a whole-genome next-generation sequencing (NGS) technique to compare wild samples to previously published genomes for two strains of *M*. *agassizii* (PS6^T^ and 723) and one strain of the closely-related microbe, *Mycoplasma testudineum* (BH29^T^) [3,15]. PS6^T^ was first collected in 1991 from a sick Mojave desert tortoise in the Las Vegas Valley, 723 was isolated from a sick gopher tortoise (*Gopherus polyphemus*) in Florida, and *M*. *testudineum* BH29^T^ was isolated from a Mojave desert tortoise but does not seem to cause respiratory disease [9,16–19].

We hypothesized that strains would show genetic diversity, as predicted by epidemiological theory and tortoise immune defenses against *M*. *agassizii*. Most of the sampled animals that were positive for *M*. *agassizii* showed no signs of disease, and our study took place in a time without epizootic conditions [9,20]. Thus, we expected that the diversity among strains would be mostly due to geographic distance. We also predicted that strains collected in 2011–2012 would show some divergence from PS6^T^, due to evolution since PS6^T^ was collected. Finally, we predicted that strains would be more closely related to PS6^T^ than to 723. We also hypothesized that we would find genetic differences in strains found in tortoises with signs of disease compared to those without. Similarly, we expected that we would find genetic differences in strains isolated from tortoise populations with a higher prevalence of disease and *M*. *agassizii* than in populations with a low signature of disease (*sensu* Sandmeier et al. [9]).

## Materials and methods

### Ethics statement

All institutional and national guidelines for the care and use of animals were followed and included permits: IACUC (00465 and 0554), Nevada Division of Wildlife (S33080), California Fish and Game (SC-007374), Utah Division of Wildlife (5COLL8886), U.S. Fish and Wildlife Service (TE030659) and National Park Service (MOJA-2012, ZION-2012-SCI-006), Arizona Game and Fish Department Permit (SP793245).

### Sample collection and culture

We collected nasal lavage samples, as previously described [13], from free-ranging Mojave and Sonoran desert tortoises in 2011–2012. Lavage samples were filtered through a 0.45 μm filter into SP4 broth and placed on ice in the field. In a sterile environment, these samples were further filtered through a 0.1 μm filter and incubated at 30˚C in a portable incubator. We used the protocol described by ATCC (https://www.atcc.org/products/all/700616.aspx), and to our knowledge, other techniques have not been verified for this organism [14,17]. In 2011, culture samples were sent on ice to collaborators at the University of Nevada, Reno for which these eight samples originated [9,13,20].

### DNA extraction and genomic sequencing

Genomic DNA was extracted using the Qiagen DNeasy Blood and Tissue protocol (Qiagen, Valencia, CA) for Gram-negative bacteria and eluted with ultrapure water. Extracted DNA was quantified using a Qiagen QIAxpert system and Picogreen assay. The presence of *Mycoplasma* spp. was verified with qPCR [21], with the assumption that either or both of *M*. *agassizii* and *M*. *testudineum* may have grown from the tortoise sample cultures. Genomic sequencing was performed using the Illumina Nextera XT DNA Library Preparation Kit (Illumina, Inc., San Diego, USA) with the Illumina NextSeq500 platform (150bp, paired-end) and up to 2 ng of DNA per sample at the Nevada Genomics Center (University of Nevada, Reno). Sequencing was achieved in a multiplex system, using dual index sequences from the Illumina Nextera XT Index kit (index 1, N701 to N715; index 2 S502 to S511). Raw sequences were deposited in the NCBI Sequence Read Archive (SRA) database under bioproject PRJNA655797.

### Data analysis

Sequencing reads were demultiplexed, assigning each read to its respective culture sample and removing barcodes. Because we detected some contamination from other bacteria, we used the bbsplit.sh script from the BBtools program [21], with default parameters, to extract *Mycoplasma*-like reads. This software extracts the sequences that are similar to a set of reference genomes (identity ≥ 35%) using a local pairwise alignment method. Three reference mycoplasma genomes were used to extract *Mycoplasma*-like reads from the raw fastq files: two *M*. *agassizii* genomes (strains PS6^T^ and 723, with NCBI accession numbers NQMN00000000 and NQNY00000000, respectively) and one *M*. *testudineum* genome (strain BH29^T^; NNCE00000000) [3,15]. The fastQC program v.0.11.8 [22] was used to evaluate the quality of the filtered fastq files.

To determine which of the three reference genomes is the most closely related to each field culture, the filtered reads were aligned to each of the three genomes using the NCBI-magicblast v1.3.0 software. For most samples, most reads matched *M*. *agassizii* PS6^T^ –the only cases in which most reads matched *M*. *agassizii* 723 or *M*. *testudineum* BH29^T^ were cases with very few *Mycoplasma*-like reads. Thus, we used *M*. *agassizii* PS6^T^ as reference in all subsequent analyses. To evaluate the accuracy of our method, we repeated our analyses using the reads previously generated in our labs for sequencing the reference *M*. *agassizii* and *M*. *testudineum* genomes [3,15].

For each sample, all filtered reads were aligned against the *M*. *agassizii* PS6^T^ genome for SNP identification. The backtrack algorithm from the Burrows-Wheeler Aligner (BWA) [23], with default parameters, was used to generate sequence alignment files (SAM files). The Sequencing Alignment/MAP software SAMtools v. 1.4 [24] was employed to generate, sort, index, and remove duplicates from the binary alignment files (BAM files). Only reads that mapped with a map quality score over 30 were retained. This map quality score threshold reduces the probability of mapping/aligning a random read to a reference genome to 0.001. For each alignment, basic statistics were calculated using the stats function from the SAMtools package. We estimated the genome coverage by averaging the per nucleotide position coverage (depth) by the PS6^T^ across all genome nucleotides. We classified our cultured samples into four categories according to their sequencing coverage: high (> 40x, n = 1), medium (25–40x, n = 2), low (2–25x, n = 5) coverage genomes, and samples with a very low number of *Mycoplasma*-like reads or very low coverage (< 2x, n = 30). De novo assembly was also performed as a complementary method to detect the presence of variants between the studied samples; to that end we used the SPAdes genome assembler v.3.9 set to default parameters.

We used the Analysis of Next Generation Sequencing Data (ANGSD) software v. 0.9 for SNP calling. The methods incorporated in ANGSD are more suitable for low and medium coverage data, because they are based on genotype likelihoods avoiding uncertainty in SNP identification typical of poor sequencing data [25]. We conducted different analyses to screen for genetic variation among our samples depending on their sequencing coverage. First, we performed pairwise comparisons between the *M*. *agassizii* PS6^T^ genome and each of the high and medium coverage samples (n = 3). For this analysis, we used the SAMtools genotype likelihood model (GL 1) implemented in ANGSD, to identify SNPs only in sites with a high probability of being polymorphic (SNP_pval < 10^−6^). In a second analysis, we performed SNP calling from a pooled dataset containing all high and medium coverage samples (n = 3). For these first two analyses, we tested several depth filters (setMinDepth), considering only sites with coverages of at least 5, 10, 15, and 20x. Finally, we conducted a similar variant identification analysis pooling all high, medium, and low coverage samples (n = 8) and both the PS6^T^ and 723 genomes. Pooling the data reduces the likelihood of sequencing errors being interpreted as SNPs. For this analysis, we used the same filters mentioned above but replaced the minimal depth filter per site by the minimal depth by sample (minIndDepth = 2). In addition, we considered only sites with more than three reads supporting a site in each sample. To avoid the false-positive identification caused by the low coverage sequencing, we only considered a variant as a SNP if it was present in more than 50% of the individuals (minInd > 5).

A maximum-likelihood phylogenetic tree was obtained using the polymorphic sites present among the high, medium, and low coverage samples and the PS6^T^ and 723 reference genomes. The tree was computed using the Molecular Evolutionary Genetics Analysis (MEGA) X software [26], using default parameters.

For the eight genome samples with low to high coverage, we evaluated whether or not the samples were collected from animals with active URTD infections, as scored by clinical signs of fresh or dried mucus in and around the nares of the animals [9]. We also determined whether there was a difference in disease or pathogen prevalence among the populations from which these eight samples originated [9,13,20]

## Results

### *Mycoplasma* spp. presence in desert tortoise isolates

*Mycoplasma agassizii* is a small, aerobic, slow-growing species, requiring weeks-to-months of culturing for visible growth. Due to its difficulty to culture, attempts to grow *M*. *agassizii* from mucus samples containing many microbes may result in false-negatives or non-pure cultures. We collected and attempted to culture *M*. *agassizii* from 92 Mojave desert tortoises and 14 Sonoran desert tortoises in 2011 and 2012. We extracted DNA from 86 cultures that appeared to have replicated and reached mid-log growth at the expected timescale. This resulted in 75 DNA samples from Mojave desert tortoises and 11 DNA samples from Sonoran tortoises that were subjected to Illumina sequencing. These samples included 67 that tested as positive for *M*. *agassizii* using qPCR after culturing. After the resulting sequencing reads were filtered using the genomes of *M*. *agassizii* (strains 723 and PS6^T^) and *M*. *testudineum* (strain BH29^T^) as reference, we obtained 214 to 688,878 *Mycoplasma*-like reads per sample in 38 samples, 37 of which were qPCR positive for *M*. *agassizii* (Tables 1 and 2 and S1). The 48 remaining samples were discarded after the filtering step due to the lack of *Mycoplasma*-like reads, which we attribute to the difficulty of culturing mycoplasmas. Eight of the samples had over 21,000 *Mycoplasma*-like reads and were used in analyses to identify SNPs, as we detailed in Table 1.

**Table 1:** Genome and read alignment statistics for reference genomes and high, medium, and low coverage field cultures.

Sample type	Host species	Sample location	Genetic population (Mojave desert tortoise)	# *Mycoplasma*-like reads	# Reads matching *M*. *agassizii* PS6^T^	# Reads matching *M*. *agassizii* 723	# Reads matching *M*. *testudineum* BH29^T^	% reads matching *M*. *agassizii* PS6^T^	Genome coverage relative to *M*. *agassizii* PS6^T^	Reads matching both PS6^T^ and 723	Reads matching both PS6^T^ and BH29^T^	% of PS6^T^ genome covered
*M*. *agassizii* PS6^T^	Mojave desert tortoise	Las Vegas Valley	California	688878	561316	16615	2302	81.5	80.6x	15488	1942	100
*M*. *agassizii* 723	Gopher tortoise	Florida	_	652230	19784	542232	2478	3.0	_	18222	441	83.1
*M*. *testudineum* BH29^T^	Mojave desert tortoise	Unknown	Unknown	874250	4963	5420	740646	0.6	_	2605	4358	4.6
Field culture (CU2012032)	Mojave desert tortoise	W. Providence Mtns	California	705988	632031	17956	1713	89.6	73.8x	17329	1534	99.9
Field culture (CU2012048)	Mojave desert tortoise	N. Coyote Springs	NE Mojave	373784	331009	9901	970	88.6	39.9x	9517	887	99.9
Field culture (CU2012019)	Mojave desert tortoise	Ord Rodman	California	212784	212833	9223	735	88.9	25.6x	8659	704	99.9
Field culture (CU2012078)	Mojave desert tortoise	South Ivanpah	Las Vegas	53372	47144	1343	150	88.3	5.8x	1295	130	93.2
Field culture (CU2012112)	Sonoran desert tortoise	Sugar Loaf	_	49132	43574	1296	123	88.7	5.4x	1240	109	91.7
Field culture (CU2012055)	Mojave desert tortoise	N. Coyote Springs	NE Mojave	25430	22469	714	71	88.4	2.7x	696	84	68.8
Field culture (CU2012105)	Sonoran desert tortoise	Sugar Loaf	_	21898	16199	756	416	74.0	2.2x	730	351	64.7
Field culture (CU2012104)	Sonoran desert tortoise	Cave Buttes	_	21688	17712	568	117	81.7	2.1x	522	48	64.0

The number of *Mycoplasma*-like reads is the number of Illumina sequencing reads that align with any of the three reference genomes. The numbers and percentages of reads matching reference genomes are based on 100% identity. Genome coverages designate the average number of reads supporting each nucleotide in the genome. The first three lines correspond to reference genomes sequenced by Weitzman et al. [3] and Alvarez-Ponce et al. [15] from ATCC strains, included here for comparison with our field cultures. California, Las Vegas, and Northeast Mojave designate distinct population segments of *G*. *agassizii*.

**Table 2:** Very low coverage genomes originated from 11 populations of Mojave desert tortoises (*Gopherus agassizii*) and one population of Sonoran desert tortoises (*Gopherus morafkai*).

Location	Host Species	Host Population within Mojave Desert	Number of cultures with sequences matching *M*. *agassizii* PS6^T^
Cave Buttes	Sonoran desert tortoise	_	1
Chemehuevi	Mojave desert tortoise	California	1
Fenner Valley	Mojave desert tortoise	California	3
Ord Rodman	Mojave desert tortoise	California	3
W. Providence Mtns	Mojave desert tortoise	California	1
S. Ivanpah	Mojave desert tortoise	Las Vegas	4
Shadow Valley	Mojave desert tortoise	Las Vegas	1
S. Coyote Springs	Mojave desert tortoise	NE Mojave	5
NW Vegas	Mojave desert tortoise	NE Mojave	3
Red Cliffs	Mojave desert tortoise	NE Mojave	3
N. Coyote Springs	Mojave desert tortoise	NE Mojave	4
Zion	Mojave desert tortoise	NE Mojave	1

The table shows samples with a sequencing coverage lower than 2x and with fewer than 21,000 Mycoplasma-like reads (n = 30). These samples showed the presence of *M*. *agassizii* but could not be included in analyses to identify SNPs due to very low coverage.

For each sample, we counted the number of reads that perfectly matched (with 100% identity) each of the three reference genomes (Table 1). We verified the validity of this analysis by confirming that reads from the reference strains correctly matched their expected genomes: most reads resulting from sequencing of the *M*. *agassizii* PS6^T^ strain (data from ref. [15]) matched the PS6^T^ genome, most reads resulting from sequencing of the *M*. *agassizii* 723 strain (data from ref. [15]) matched the 723 genome, and most reads resulting from sequencing of the *M*. *testudineum* BH29^T^ strain (data from ref. [3]) matched the BH29^T^ genome (Table 1). The three reference strains/species also showed significant overlap among sequence reads (some reads perfectly matched two or all three genomes), indicating that there are conserved regions among these three genomes (Table 1), as expected from the fact that they are closely related [3,15]. Thus, some of the reads derived from sequencing one of the reference genomes matched not only that genome, but also one or the two other reference genomes.

For all of the 14 samples with more than 5,752 *Mycoplasma*-like reads (Tables 1 and S2), the numbers of reads matching the *M*. *agassizii* PS6^T^ genome were higher than those matching the *M*. *agassizii* 723 genome, and much higher for either *M*. *agassizii* genome than for the *M*. *testudineum* BH29^T^ genome (Table 1). This indicates that the field cultures are more closely related to *M*. *agassizii* PS6^T^ than to the other strain of *M*. *agassizii* or to *M*. *testudineum*. In the 24 samples with fewer than 5,752 total *Mycoplasma*-like reads, we found that 6 had more reads matching *M*. *agassizii* 723 than reads matching *M*. *agassizii* PS6^T^, but all had fewer reads that matched *M*. *testudineum*.

**Fig 1:**
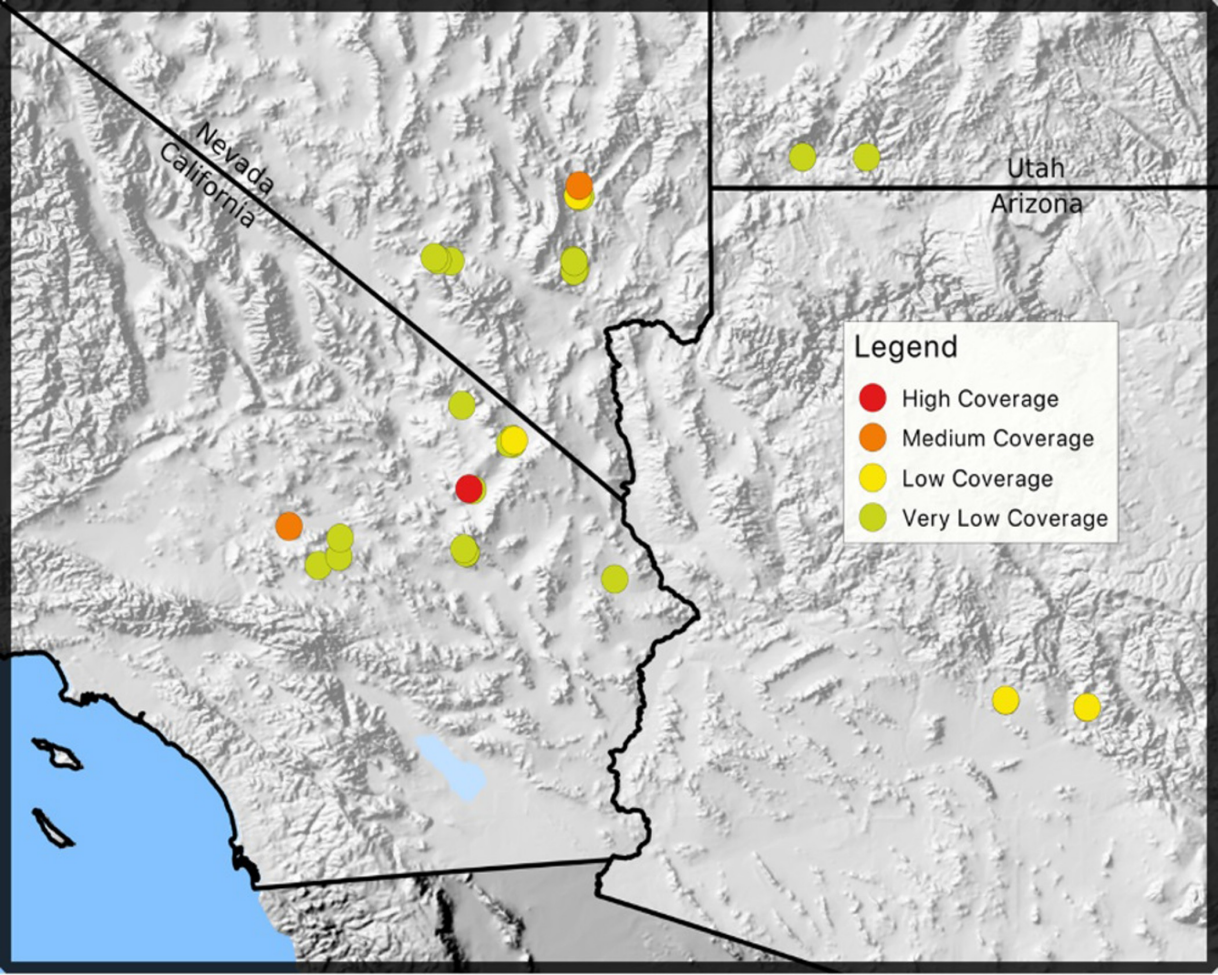
Locations in the Mojave and Sonoran Deserts where Mycoplasma spp. isolates were collected. Locations are color-coded based on genome coverage from next-generation sequencing: high (>40x), medium (>25x), low (>2x), and very low (<2x). The map was created with data from the USGS National Map Viewer.

Given that all higher-quality field culture sequencing datasets matched the *M*. *agassizii* PS6^T^ genome better than the *M*. *agassizii* 723 or *M*. *testudineum* BH29^T^ genomes (Table 1), all subsequent analyses were conducted using the PS6^T^ genome as reference. As depicted in Fig 1 and Table 1, one sample had high coverage (61x), two samples had medium coverage (25x and 39x), five samples had low coverage (5x to 2x), and the remaining 30 samples had very low coverage (lower than 2x; Tables 2 and S1 and Fig 1). Low coverage samples still indicated similarity to *M*. *agassizii* PS6^T^ and represent diverse tortoise populations across the Mojave and Sonoran Deserts (S1 Table and Fig 1).

### Lack of genetic variation among *Mycoplasma agassizii* isolates from desert tortoises

**Fig 2:**
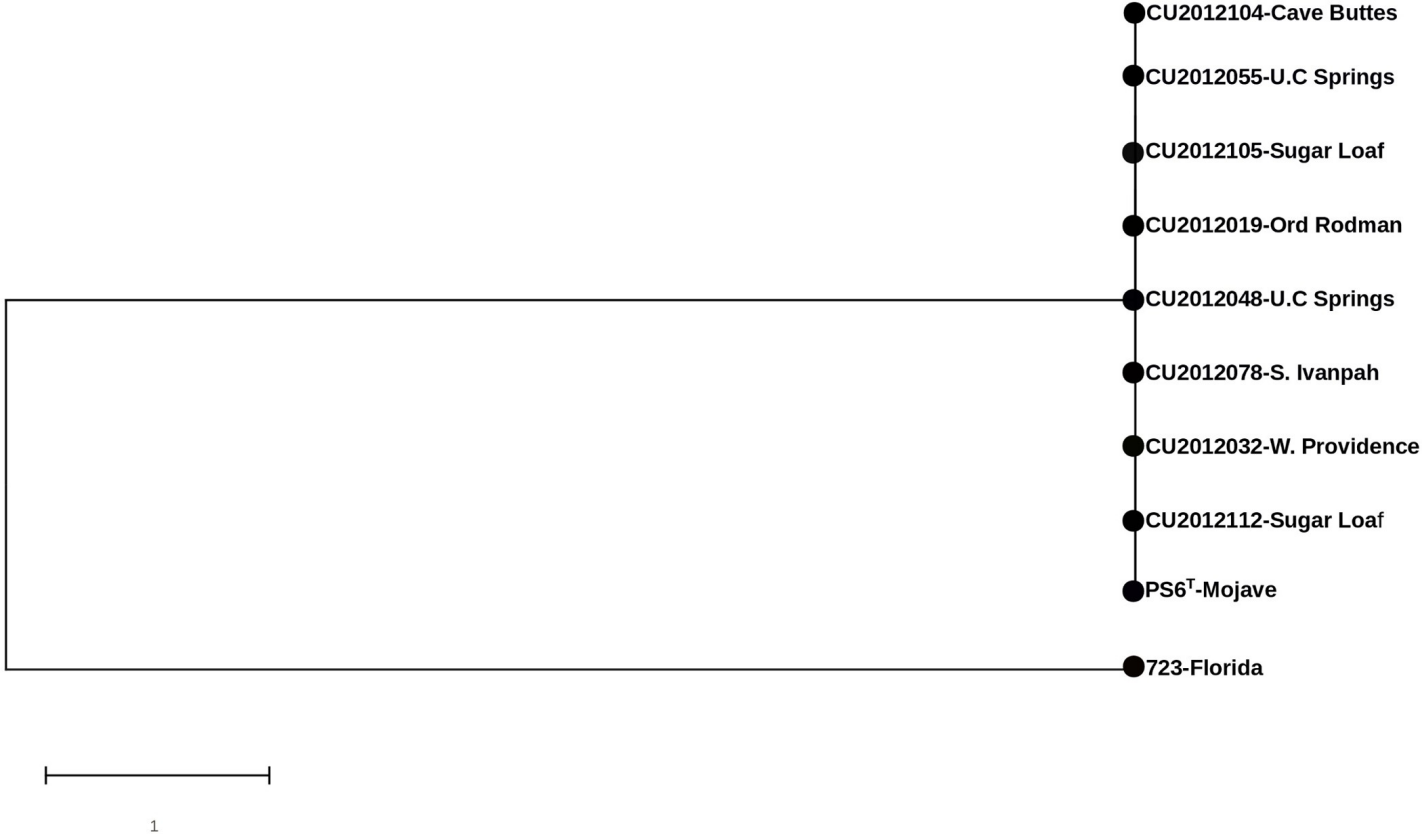
Maximum likelihood phylogenetic tree of *Mycoplasma agassizii* from eight samples with low to high coverage and the PS6^T^ and 723 strains based on 1373 SNPs.

We aligned all the reads resulting from the high (n = 1), medium (n = 2) and low (n = 5) coverage samples to the reference *M*. *agassizii* PS6^T^ genome for SNP identification. The SNP calling for these eight samples covered between 54% and 99.9% of the PS6^T^ genome used as reference. Pairwise comparisons between the reference *M*. *agassizii* PS6^T^ genome and each of the high and medium coverage sampled genomes did not identify any SNPs. Similarly, analysis of a pooled dataset containing all high and medium coverage genomes (n = 3) did not reveal any SNPs. When we used more stringent criteria for SNP calling, but also included the 5 low coverage genomes (n = 8), we still did not identify any SNPs. The absence of SNPs was confirmed by visual inspection of the alignments using the Integrative Genomics Viewer program (IGV v2.3; ref. [27])–no potential SNP was supported by more than 2 reads. The lack of variation was confirmed for the three samples with the higher number of reads. To that end, we performed *de novo* assembling and compared the resulting contigs to the PS6^T^ genome using the reordering function from the ABACAS algorithm [28]. Comparison of the genomes of *M*. *agassizii* strains PS6^T^ and 723 and our samples, however, resulted in 1373 SNPs, which resulted from differences specific to *M*. *agassizii* 723. A maximum likelihood phylogenetic tree of all our high, medium and low coverage samples and the two *M*. *agassizii* reference genomes is shown in Fig 2.

Table 3 depicts the disease status of these same eight samples and their respective populations, previously described in Weitzman et al. [13,20] and Sandmeier et al. [9]. None of these tortoises exhibited signs of disease at the time of sampling. Within the Mojave Desert, these eight tortoise populations represent an average level of clinical disease from all 24 populations that were surveyed over this same time period (0–25% prevalence of disease; refs. [9,13]). In addition, they span almost the entire range of prevalence of *M*. *agassizii* also quantified in 24 populations across the range (0–90% prevalence of *M*. *agassizii*; refs. [9,13]).

**Table 3:** Disease status (clinical signs and *Mycoplasma agassizii*) of individuals and their respective populations from which high-quality genome sequences were obtained (see Table 1).

Sample	Host species	Tortoise population*	Signs of disease*	*M*. *agassizii* qPCR (Ct values)*	*M*. *testudineum* qPCR (Ct values)*	Prevalence of *M*. *agassizii in the* population (%)* **	Prevalence of disease in the population (%)*
Field culture (CU2012032)	Mojave desert tortoise	W. Providence Mtns	None	Negative	Negative	31	12
Field culture (CU2012048)	Mojave desert tortoise	N. Coyote Springs	None	35.31	Negative	65	14
Field culture (CU2012019)	Mojave desert tortoise	Ord Rodman	None	37.89	Negative	74	11
Field culture (CU2012078)	Mojave desert tortoise	S. Ivanpah	None	Negative	Negative	56	13
Field culture (CU2012112)	Sonoran desert tortoise	Sugar Loaf	None	Negative	Negative	80	4
Field culture (CU2012055)	Mojave desert tortoise	N. Coyote Springs	None	39.1	Negative	65	14
Field culture (CU2012105)	Sonoran desert tortoise	Sugar Loaf	None	Negative	Negative	80	4
Field culture (CU2012104)	Sonoran desert tortoise	Cave Buttes	None	Negative	Negative	38	0

* Previously published in Weitzman et al. [13,20]; Sandmeier et al. [9].

** Prevalence determined from uncultured DNA samples, tested by qPCR.

## Discussion

To our knowledge, this is the first study that has successfully cultured and described *M*. *agassizii* from predominantly subclinical tortoises across the entire range of Mojave desert tortoises as well as some Sonoran desert tortoise locations. Despite a limited number of samples with deep coverage, we used conservative techniques to identify samples as identical or nearly identical to the type culture isolate PS6^T^. This was surprising, given the high genetic variation in most species of mycoplasmas [29]. Disease in tortoises has been shown to be caused by both recrudescence and transmission, which would lead to predictions of evolution within the host, transmission of variable strains among individuals, and high genetic variation [10], a pattern seen in other chronic mycoplasmal diseases [30,31]. Similarly, at the time that PS6^T^ was isolated from tortoises in the Las Vegas Valley, a variety of cultured strains were identified from different tortoises based on phenotypic characteristics [17]. However, it is important to emphasize that we filtered all our field samples prior to culturing in an effort to reduce contamination. Brown et al. [17] showed that not all isolates cultured equally well after passage through filters. Below, we discuss two alternate interpretations of our data. (1) PS6^T^ is indeed the most common genetic strain of *M*. *agassizii* in Mojave and possibly also in Sonoran desert tortoises. (2) PS6^T^ cultures more readily than other strains, and we thus detected this strain in many of our sampled tortoises. We compare the patterns described with more well-characterized host–mycoplasma systems in agricultural animals and humans. Regardless of this unresolved pattern, we found that Mojave desert tortoises seem to differ in their endemic *M*. *agassizii* from strain 723 that originated in gopher tortoises [17]. Finally, we suggest other tools for studying this disease given current limitations in reliable culturing techniques.3.1. *Mycoplasma* spp. presence in desert tortoise isolates.

### Alternate explanations for patterns of little genetic variation

Eight samples from diverse locations (Fig 1) had >21,000 *Mycoplasma*-like sequencing reads, or a coverage greater than 2x (Tables 1 and S2). In these sequences we found that most reads matched PS6^T^ and not 723 (Tables 1 and S2). Furthermore, most of the reads that did match 723 also matched PS6^T^, indicating that they fall within conserved regions of these two similar genomes [15]. Strikingly, we identified no SNPs, even though Weitzman et al. [13] found a very low frequency of SNPs in conserved regions of the genome in uncultured samples from these same populations (nonetheless, the samples used by Weitzman et al. do not overlap with the ones used here). However, Weitzman et al. [13] conducted Sanger sequencing on 25–51 samples, representing 16S rRNA, the 16-23S intergenic spacer region, and RNA polymerase ß subunit. Therefore, our study corroborates previous results that even in ribosomal regions, *M*. *agassizii* has relatively little genetic variation–even though mycoplasmas generally have much higher variation in ribosomal DNA than is found in other organisms [6,7,32].

Because we observed a very low incidence of disease during our three-year survey of Mojave and Sonoran desert tortoises [9,20], we do not attribute the pattern of little genetic diversity to clonal spread of a virulent mycoplasmal strain (e.g., ref. [33]). Although PS6^T^ was isolated from a wild tortoise with signs of URTD in Las Vegas in 1991 [17,34], it is of unknown virulence and is not the highly virulent strain that was used in an earlier infection experiment that led to rapid, consistent disease in all exposed tortoises [14]. To our knowledge, that isolate has not been preserved or genetically sequenced, despite its crucial role in identifying *M*. *agassizii* as the etiological agent of URTD by the almost complete fulfillment of Koch’s postulates [14]. Of course, current genetic technology did not exist at the time when outbreaks of disease were most severe (1989–1991) and strains were intentionally cultured from epizootic areas for experimental investigations of URTD’s etiology (reviewed in refs. [35,36]).

If PS6^T^ is the most dominant strain of *M*. *agassizii* in the Mojave and Sonoran Deserts, that would suggest stabilizing selection or low mutation rates, in particular because there has been so little change over time (18 years since PS6^T^ was originally collected) and space (the extent of the Mojave desert; Fig 1). Interestingly, other mycoplasmal diseases show more genetic variation but also often do not show evolutionary patterns related to geography, time, or disease status of individuals [30,37,38]. More similar to our own results, Xiao et al. [5] found high genetic similarity of samples of another chronic respiratory pathogen, *M*. *pneumoniae*, in human populations. Such patterns could occur in systems where low virulence or selection for a constant phenotype results in higher fitness for the pathogen. However, high similarity between *M*. *pneumoniae* genomes is associated to their geographical distribution and could also depend on the circulation period. For instance, a recent study detected up to 5% variability between genomes from different circulation times and sampling locations across South Korea [39]. Low mutation rates have not been documented for other species of mycoplasmas, and the class Mollicutes shows some of the highest mutation rates of any group of prokaryotes [31]. In these other species, high mutation rates have been attributed to loss of DNA-repair mechanisms [6,7]. If *M*. *agassizii* truly does have low mutation rates, it would be interesting to test whether selection pressure could have led to more stable DNA replication and repair in this species.

Indeed, *M*. *agassizii* does not seem to be very virulent during average or stable environmental conditions. For example, transmission of *M*. *agassizii* between tortoises is so slow that it requires 24–48 hours of close contact [8]. In addition, the majority of tortoises that test positive for *M*. *agassizii* show no signs of disease, have very low microbial loads, and seem to be chronic carriers of the microbe [8,10]. Because tortoise adaptive immune responses are ineffective at clearing *M*. *agassizii*, such host responses are unlikely to act as a selective pressure for higher virulence or evasion of the host immune system [1,2]. In fact, it may lead to higher fitness of the pathogen to remain relatively avirulent and increase the likelihood of transmission to another host [2]. However, PS6^T^ was initially collected from a diseased individual, and it may represent a strain that can become more virulent or transmit more easily at higher loads [8,9]. Stabilizing selection or selection for small phenotypic changes may be part of maintaining chronic infections and the suspected long co-evolution between this host and its pathogen [2,12].

The alternate, not mutually-exclusive explanation is that multiple strains, including PS6^T^, are maintained in individual tortoises or populations, but we lacked the tools to identify them. Because our field samples had low initial cell numbers, we needed to first culture the samples before extracting and sequencing DNA. However, recent research using genetic techniques in the absence of culturing clinical samples shows that identification of multi-strain infections and a greater diversity of strains is becoming increasingly common [40,41]. Culturing microbial samples often biases results toward the fastest growing strain, thus leading to erroneous conclusions about the true natural diversity of strains [42,43]. Our use of two filtering steps and the culture conditions recommended specifically for PS6^T^ may have caused us to select for this strain from our wild samples [17]. While contamination of cultures could also explain recovery of the same genetic strain, samples were collected, cultured, and frozen at disparate and non-overlapping times. Similarly, they were not exposed to the cultures of the type strain PS6^T^.

### General patterns and future directions

We found two other patterns in our dataset. First, we did not detect any sample that clearly identified more closely to 723 than to PS6^T^. Furthermore, the reads that did align with the strain originally isolated from a gopher tortoise from Sanibel Island, Florida (strain 723) were from regions of the genome that are conserved between the two isolates (Tables 1 and S2). This suggests that, similar to other mycoplasmas that infect multiple species, there is strain variation among host species [44–46]. However, we found no variation in *M*. *agassizii* from Mojave and Sonoran desert tortoises. Whether this is due to closer genetic similarity between the two desert tortoise species versus gopher tortoises, or due to geographic distance–with PS6^T^ predominant in the West and 723 or another strain predominant in the East–remains to be determined.

The second pattern is that all of the eight best-quality sequencing datasets were obtained from tortoises without any signs of disease (Table 3). These tortoises spanned populations with moderate disease prevalence and wide variation of *M*. *agassizii* prevalence, representative of values found throughout the region (Table 3; refs. [9,13,20]). Thus, PS6^T^ is found across the Mojave and Sonoran deserts, regardless of levels of higher or lower disease in distinct populations. If strains of *M*. *agassizii* are homogenously spread across the two deserts, then it seems unlikely that pathogen genetics predicts the variable patterns of disease observed in tortoises [35,36]. Given recent evidence of differences in Mojave tortoise immune parameters and their relationship to load of *M*. *agassizii* and prevalence of disease across populations, it is likely that the drivers of disease reside primarily in the host immune function, triggered when *M*. *agassizii* load is high [9,13,20]. Experimental studies are needed to tease apart these factors, and possibly relate them to different environmental conditions. For example, both host and pathogen factors have been shown to drive mycoplasmal disease in house finches. Differences in virulence of *M*. *gallisepticum* and not host immunity appear to drive prevalence of disease in some wild populations [47]. Additionally, it has also been shown that host tolerance and resistance have evolved in response to pathogen pressure [4,48,49].

Finally, we suggest using additional, culture-independent genetic tools to answer the question of whether mixed strain infections are common in the respiratory tract of tortoises. The fact that researchers can extract only small quantities of this microbe from the nares of live animals is a characteristic that is shared with many other wildlife diseases [50]. Similarly, the fastidious nature of *M*. *agassizii* in culture is also shared by many mycoplasmas and other microbes [11,51]. We suggest that we can use the published genomes of *M*. *agassizii* and *M*. *testudineum* to identify genetic regions with expected high rates of variability (e.g., refs. [3,15,44]). In particular, we may see differences in virulence genes and genes associated with growth rates across isolates [17]. Like most other species of mycoplasma, *M*. *agassizii* has only one copy of 16S rRNA, and the qPCR for this gene can thus be used to quantify the number of cells within a sample [6,52]. If we then create a series of qPCR assays for potential virulence and growth factors in PS6^T^, we can test for a gain or loss of those genes per *M*. *agassizii* cell in specimens obtained from wild animals. Similar assays have been used to detect loss and gain of virulence genes in human and livestock diseases [53–55]. Gain and loss of genes may be common in mycoplasmas [56], and quantifying such changes will allow us to test whether tortoises have mixed-strain infections. Quantitative PCR is especially powerful in analyzing samples with relatively low quantities of DNA, but has only recently been used for quantifying disease and genetic differences in and among samples, (e.g., refs. [52,55]).

## Conclusions

In conclusion, we verified that *M*. *agassizii* strain PS6^T^ exists in subclinical Mojave and Sonoran desert tortoises. This pattern suggests that enzootic, not epizootic, disease conditions may be the norm across these populations, at least in recent times. However, we also suggest that culturing samples may have skewed our data and will create a similar problem in other research. We suggest that qPCR may be an alternate approach to explore true diversity of mycoplasmal infections within uncultured samples taken from both subclinical and diseased wild animals.

## Supporting information

S1 Table Results of qPCR reactions for cultured field samples using *Mycoplasma agassizii* primers and probe. (PDF)

S2 Table Genome and read alignment statistics for reference genomes with very low coverage field cultures. (PDF)
