# Supplementary material for: Mycoplasma agassizii, an opportunistic pathogen of tortoises, shows very little genetic variation across the Mojave and Sonoran Deserts
Source: PLoS One. 2021 Feb 3;16(2):e0245895. doi: 10.1371/journal.pone.0245895 (PMC7857612; doi:10.1371/journal.pone.0245895)
Supplement: S1 Table — (PDF) [file pone.0245895.s001.pdf]

Table S1. Results of qPCR reactions for cultured field samples using *Mycoplasma agassizii* primers and probe.

Sample	Host species	Host location	Average cycle threshold (Ct) values
**CU2011008**	*Gopherus agassizii*	Lower Coyote Springs	29.8
**CU2011010**	*Gopherus agassizii*	Lower Coyote Springs	29.9
**CU2011013**	*Gopherus agassizii*	Lower Coyote Springs	30.2
**CU2011014**	*Gopherus agassizii*	Lower Coyote Springs	30.3
**CU2011015**	*Gopherus agassizii*	Lower Coyote Springs	NA
**CU2011041**	*Gopherus agassizii*	W. Providence Mtns	30.2
**CU2012019**	*Gopherus agassizii*	Ord Rodman	31.0
**CU2012024**	*Gopherus agassizii*	Ord Rodman	30.3
**CU2012025**	*Gopherus agassizii*	Ord Rodman	30.0
**CU2012028**	*Gopherus agassizii*	Ord Rodman	32.9
**CU2012032**	*Gopherus agassizii*	W. Providence Mtns	29.7
**CU2012035**	*Gopherus agassizii*	Fenner Valley	29.6
**CU2012036**	*Gopherus agassizii*	Fenner Valley	32.0
**CU2012037**	*Gopherus agassizii*	Fenner Valley	33.3
**CU2012038**	*Gopherus agassizii*	NW Vegas	30.0
**CU2012041**	*Gopherus agassizii*	NW Vegas	30.5
**CU2012044**	*Gopherus agassizii*	S. Ivanpah	34.3
**CU2012045**	*Gopherus agassizii*	S. Ivanpah	31.7
**CU2012046**	*Gopherus agassizii*	S. Ivanpah	29.0
**CU2012047**	*Gopherus agassizii*	S. Ivanpah	31.7
**CU2012048**	*Gopherus agassizii*	Upper Coyote Springs	27.7
**CU2012051**	*Gopherus agassizii*	Upper Coyote Springs	33.4
**CU2012055**	*Gopherus agassizii*	Upper Coyote Springs	34.4
**CU2012056**	*Gopherus agassizii*	Upper Coyote Springs	35.6
**CU2012057**	*Gopherus agassizii*	Upper Coyote Springs	35.9
**CU2012063**	*Gopherus agassizii*	NW Vegas	34.4
**CU2012078**	*Gopherus agassizii*	S. Ivanpah	31.3
**CU2012084**	*Gopherus agassizii*	Chemehuevi	32.9
**CU2012088**	*Gopherus agassizii*	Shadow Valley	33.3
**CU2012102**	*Gopherus morafkai*	Cave Buttes	34.1
**CU2012104**	*Gopherus morafkai*	Cave Buttes	31.6
**CU2012105**	*Gopherus morafkai*	Sugar Loaf	33.9
**CU2012112**	*Gopherus morafkai*	Sugar Loaf	32.5
**CU2012118**	*Gopherus agassizii*	Red Cliffs	28.8
**CU2012119**	*Gopherus agassizii*	Red Cliffs	29.0
**CU2012121**	*Gopherus agassizii*	Red Cliffs	28.1
**CU2012122**	*Gopherus agassizii*	Red Cliffs	33.5
**CU2012126**	*Gopherus agassizii*	Zion	30.8
