# Supplementary material for: Mycoplasma agassizii, an opportunistic pathogen of tortoises, shows very little genetic variation across the Mojave and Sonoran Deserts
Source: PLoS One. 2021 Feb 3;16(2):e0245895. doi: 10.1371/journal.pone.0245895 (PMC7857612; doi:10.1371/journal.pone.0245895)
Supplement: S2 Table — (PDF) [file pone.0245895.s002.pdf]

**Table S2.** Genome and read alignment statistics for reference genomes with very low coverage field cultures.

| Sample type | Host species | Sample location | Genetic population (Mojave desert tortoise) | # *Mycoplasma*-like reads | # Reads matching *M. agassizii* PS6$^T$ | # Reads matching *M. agassizii* 723 | # Reads matching *M. testudineum* BH29$^T$ | % reads matching *M. agassizii* PS6$^T$ | Genome coverage relative to *M. agassizii* PS6$^T$ | Reads matching both PS6$^T$ and 723 | Reads matching both PS6$^T$ and BH29$^T$ | % of PS6$^T$ genome covered |
|---|---|---|---|---|---|---|---|---|---|---|---|---|
| Field culture (CU2012037) | Sonoran desert tortoise | Cave Buttes | NA | 13246 | 11369 | 288 | 37 | 85.8 | 1.3 | 281 | 48 | 38 |
| Field culture (CU2012102) | Mojave desert tortoise | Fenner Valley | California | 11350 | 10105 | 294 | 40 | 89.1 | 1.2 | 285 | 28 | 47.2 |
| Field culture (CU2012047) | Mojave desert tortoise | S. Ivanpah | Las Vegas | 8316 | 7224 | 220 | 29 | 86.9 | 0.9 | 211 | 30 | 38 |
| Field culture (CU2012088) | Mojave desert tortoise | Shadow Valley | Las Vegas | 6876 | 5688 | 181 | 63 | 82.5 | 0.7 | 168 | 16 | 29.2 |
| Field culture (CU2012044) | Mojave desert tortoise | S. Ivanpah | Las Vegas | 5752 | 5054 | 165 | 19 | 87.9 | 0.6 | 81 | 45 | 31 |
| Field culture (CU2012051) | Mojave desert tortoise | N. Coyote Springs | NE Mojave | 6034 | 4398 | 241 | 120 | 72.9 | 0.6 | 228 | 16 | 18.6 |
| Field culture (CU2012045) | Mojave desert tortoise | S. Coyote Springs | NE Mojave | 3256 | 2730 | 129 | 34 | 49.4 | 0.3 | 123 | 84 | 16.9 |
| Field culture (CU2012063) | Mojave desert tortoise | W. Providence Mtns | California | 2788 | 2221 | 96 | 27 | 79.7 | 0.3 | 92 | 12 | 11.6 |
| Field culture (CU2012028) | Mojave desert tortoise | NW Vegas | Las Vegas | 2670 | 2163 | 78 | 28 | 81 | 0.3 | 75 | 24 | 9.7 |
| Field culture (CU2012084) | Mojave desert tortoise | S. Ivanpah | Las Vegas | 4274 | 1217 | 239 | 202 | 28.5 | 0.3 | 227 | 12 | 7.9 |
| Field culture (CU2011013) | Mojave desert tortoise | Chemehuevi | California | 3244 | 575 | 316 | 38 | 2.8 | 0.2 | 0 | 166 | 0.2 |

| | | | | | | | | | | | | |
|---|---|---|---|---|---|---|---|---|---|---|---|---|
| Field culture (CU2012036) | Mojave desert tortoise | Ord Rodman | California | 1712 | 1400 | 58 | 20 | 36.1 | 0.2 | 58 | 0 | 0.2 |
| Field culture (CU2011014) | Mojave desert tortoise | S. Coyote Springs | NE Mojave | 3704 | 979 | 809 | 177 | 11 | 0.2 | 694 | 7 | 8.2 |
| Field culture (CU2012056) | Mojave desert tortoise | Zion | NE Mojave | 1740 | 1456 | 53 | 13 | 55.1 | 0.2 | 53 | 144 | 0.3 |
| Field culture (CU2012025) | Mojave desert tortoise | S. Coyote Springs | NE Mojave | 4966 | 447 | 562 | 522 | 2.5 | 0.1 | 0 | 12 | 9.6 |
| Field culture (CU2012126) | Mojave desert tortoise | Fenner Valley | California | 2892 | 233 | 226 | 176 | 8.1 | 0.1 | 213 | 137 | 0.4 |
| Field culture (CU2012122) | Mojave desert tortoise | N. Coyote Springs | NE Mojave | 1034 | 867 | 26 | 3 | 83.8 | 0.1 | 0 | 1 | 0.5 |
| Field culture (CU2011008) | Mojave desert tortoise | Ord Rodman | California | 1648 | 203 | 300 | 86 | 32.6 | 0.1 | 0 | 0 | 6 |
| Field culture (CU2011010) | Mojave desert tortoise | S. Coyote Springs | NE Mojave | 3688 | 537 | 201 | 65 | 14.6 | 0.1 | 0 | 0 | 0.2 |
| Field culture (CU2012035) | Mojave desert tortoise | Fenner Valley | California | 729 | 323 | 298 | 84 | 14.7 | 0.1 | 169 | 26 | 0.2 |
| Field culture (CU2012057) | Mojave desert tortoise | N. Coyote Springs | NE Mojave | 1768 | 117 | 117 | 127 | 6.6 | 0.1 | 111 | 91 | 0.4 |
| Field culture (CU2012024) | Mojave desert tortoise | Ord Rodman | California | 1844 | 244 | 181 | 125 | 13.2 | 0.1 | 165 | 91 | 0.2 |
| Field culture (CU2012046) | Mojave desert tortoise | S. Ivanpah | Las Vegas | 1308 | 141 | 153 | 36 | 6.3 | 0.1 | 135 | 28 | 0.7 |
| Field culture (CU2012121) | Mojave desert tortoise | Red Cliffs | NE Mojave | 771 | 130 | 147 | 97 | 7.7 | 0 | 124 | 26 | 0.2 |
| Field culture (CU2011041) | Mojave desert tortoise | N. Coyote Springs | NE Mojave | 828 | 412 | 422 | 383 | 49.2 | 0 | 72 | 359 | 0.2 |

| | | | | | | | | | | | | |
|---|---|---|---|---|---|---|---|---|---|---|---|---|
| Field culture (CU2012041) | Mojave desert tortoise | NW Vegas | Las Vegas | 828 | 83 | 74 | 42 | 9.9 | 0 | 72 | 34 | 0.1 |
| Field culture (CU2012118) | Mojave desert tortoise | Red Cliffs | NE Mojave | 1330 | 202 | 131 | 24 | 15.2 | 0 | 120 | 15 | 0.3 |
| Field culture (CU2012038) | Mojave desert tortoise | NW Vegas | Las Vegas | 880 | 147 | 69 | 65 | 16.7 | 0 | 63 | 48 | 0.7 |
| Field culture (CU2012119) | Mojave desert tortoise | Red Cliffs | NE Mojave | 298 | 59 | 60 | 13 | 18.9 | 0 | 54 | 11 | 0.2 |
| Field culture (CU2011015) | Mojave desert tortoise | S. Coyote Springs | NE Mojave | 214 | 20 | 17 | 13 | 8.9 | 0 | 17 | 13 | 0.1 |

Genetic populations of Mojave desert tortoises based on Hagerty and Tracy 2010 [1].

## References


1. Hagerty, B.E.; Tracy, C.R. Defining population structure for the Mojave desert tortoise. *Conservation genetics* **2010**, *11*, 1795-1807.
